# CuFe_2_S_3_ as electrode material for Li-ion batteries

**DOI:** 10.1039/c8ra03840f

**Published:** 2018-07-26

**Authors:** Emmanuel Anger, Antoine Maignan, Tristan Barbier, Valerie Pralong

**Affiliations:** Normandie Univ, ENSICAEN, UNICAEN, CNRS, CRISMAT 14000 Caen France valerie.pralong@ensicaen.fr

## Abstract

Electrochemical performances of the isocubanite CuFe_2_S_3_ tested as electrode material for Li-ion batteries have been investigated. A first discharge capacity of 860 mA h g^−1^ shows a conversion process leading to Li_2_S, copper and iron nanoparticles. Interestingly, a reversible capacity of 560 mA h g^−1^ at 1.5 V is demonstrated with good cyclability up to 30 cycles.

## Abstract 2

Electrochemical performances of the isocubanite CuFe_2_S_3_ tested as electrode material for Li-ion batteries have been investigated.

## Graphical abstract

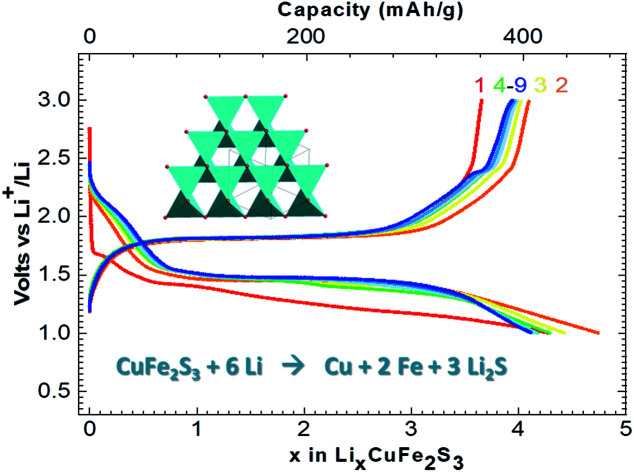

Because of their low cost and high theoretical capacity, metal sulfides are considered as one potential future electrode material for Li-ion batteries (LIBs). Therefore, numerous materials containing sulfur have been studied in the last decades as cathode materials such as MnS,^1^ Cu_*x*_S,^2^ CoS,^3^ NiS,^4^ and Fe_*x*_S.^5^ Moreover, some metal sulfides have also been reported as anode materials such as SnS_2_ (ref. 6) and ZnS.^7^ Their reductions happen through a conversion process at low working voltage (under 1 V *vs.* Li^+^/Li) leading to formation of lithium sulfide and native metal.^8^

Among the conversion materials based on transition metal sulfur, copper and iron are the most cost effective, light and non-toxic elements that one can find. For these reasons, Cu_*x*_S^2,9,10^ and Fe_*x*_S^5,11,12^ have been well studied in the last few decades using either polymer or liquid electrolytes. From these studies, we know that the reduction of these transition metal sulfides forms nanoparticles of metal and lithium sulfides through a conversion process.^8^ Therefore, recent articles have been focusing on improving the electrochemical performance of metal sulfides by using hydrothermal,^11^ sol–gel^13^ or ball-milling^14^ synthetic methods.

Copper/iron sulfides are interesting electrode materials because of their higher electrical conductivity and electrochemical activity compared to monometal sulfides.^15^ In recent years, only a few studies have been reported on chalcopyrite CuFeS_2_, first as a cathode in a primary battery^16^ then as an anode and cathode in a secondary battery.^15,17,18^ Different synthetic routes from solvothermal to nanocrystal growth and different electrolytes have proven the potential of chalcopyrite as electrode material for lithium batteries.

Herein, we report the electrochemical performance of the cubic cubanite, so-called isocubanite CuFe_2_S_3_, as the first ternary metal sulfide electrode material for lithium batteries. This Li/CuFe_2_S_3_ system exhibits a high reversibility (up to 560 mA h g^−1^ at C/20/Li) and a good cyclability over 30 cycles. *Ex situ* X-ray diffraction (XRD)††The product was characterized by XRD using a Philips X'Pert diffractometer with Bragg–Brentano geometry (CuK_α1,2_ radiation). Note that due to their instability in air, the reduced phases XRD patterns were registered under vacuum using a chamber attached to a Bruker D8 diffractometer. Electrochemical characterizations of CuFe_2_S_3_ have been performed in Swagelok cells. Metallic lithium (Aldrich, 99,9%) has been used as negative electrode, LP30 from Merck [1 M LiPF_6_ in an ethylene carbonate/dimethyl carbonate 1 : 1 (w/w) Selectipur] was used as the electrolyte, and the positive electrode was constituted of approximately 10 mg of a mixture of the active material with 50% weight of carbon (acetylene black). The electrochemical cells were cycled at constant current between 1.2 and 3.0 V at different galvanostatic rates on a VMP II potentiostat/galvanostat (Biologic SA, Claix, France) at room temperature. Potentiostatic intermittent titration technique (PITT) measurements were conducted using a potential step of 10 mV limited by a minimum current equivalent to a C/10 galvanostatic rate. measurements allow a better understanding of the electrochemical mechanism.

The cubanite CuFe_2_S_3_ phase crystallizes within an orthorhombic structure (space group: *Pcmn*), with *a* = 6.467 Å, *b* = 11.110 Å and *c* = 6.230 Å.^19^ When the orthorhombic CuFe_2_S_3_ is heated above 473 K, an irreversible structural transition occurs and CuFe_2_S_3_ adopts a cubic structural type (space group: *F*4̄3*m*, *a* = 5.296 Å). It should be noticed that cubanite and its cubic polymorph isocubanite are usually found in their natural states intimately intergrown with other sulfides such as chalcopyrite and pyrrhotite. Synthesis of isocubanite CuFe_2_S_3_ has been first reported by S. Pareek *et al.*^20^ In this paper, we chose a synthetic protocol recently described by Barbier *et al.*^21^ Following precursors: Cu (99.0%), Fe (99.5%), and S (99.5%) from Alfa Aesar, were mixed in the appropriate ratio. After sealing the cold pressed powder in a silica tube, the latter was heated at 873 K for 48 h. The room temperature powder X-ray diffraction pattern of CuFe_2_S_3_ (depicted in Fig. 1) shows that CuFe_2_S_3_ crystallizes within the cubic form. Rietveld refinements were therefore carried out using *F*4̄3*m* space group. However, extra peaks and peak shoulders which may be attributed to the chalcopyrite phase can be observed (inset Fig. 1). Thus, from the refinements, the CuFe_2_S_3_ isocubanite (around 72 wt% – 63 at%) is the majority phase, while the minority one is the chalcopyrite (around 28 wt% – 37 at%). The structural refinement leads to unit cell parameters *a* = 5.3018(1) Å and *a* = 5.2927(3) Å, *c* = 10.4340 Å for the isocubanite and chalcopyrite phases, respectively. The aforementioned unit cell parameters are close to those reported in the literature and then confirms their good crystallinity.^22^ The isocubanite structure can be described as a tetragonal close-packed stacking of S^2−^ anions, which occupy the 4*a* (0, 0, 0) crystallographic site while Cu and Fe cations are randomly distributed over the two structurally equivalent tetrahedral sites 4*c* (1/4, 1/4, 1/4) and 4*d* (3/4, 3/4, 3/4). Different Rietveld refinements were therefore performed with both 4*c* and 4*d* crystallographic sites, and best result through low isotropic displacement parameters and reliability factors (*χ*^2^ = 2.303 and *R*_Bragg_ factor = 6.98%) was obtained with all Cu and Fe atoms on the 4*d* crystallographic site. Although the obtained sample is intergrown with chalcopyrite; as previously studied, the chalcopyrite phase forms submicronic domains, this isocubanite sample is well crystallized.^21^ Note that the average particle size is about 1–2 μm without any particular shape.

**Fig. 1:**
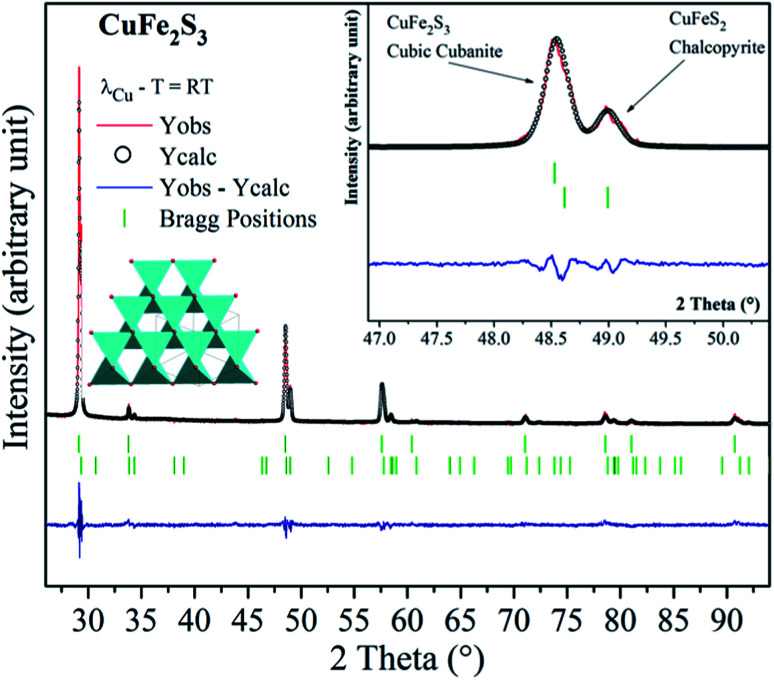
Powder X-ray diffraction pattern of CuFe_2_S_3_ isocubanite. Vertical bars, respectively, indicate the Bragg peak positions corresponding to the chalcopyrite CuFeS_2_ (bottom green bars – space group: I4̄2d no. 122) and to the cubic cubanite so-called isocubanite CuFe_2_S_3_ (top green bars space group: *F*4̄3*m* no. 216). Inset shows a zoomed-in portion of the aforementioned figure, showing the coexistence of the CuFe_2_S_3_ isocubanite and CuFeS_2_ chalcopyrite.

The charge–discharge profiles of Li/CuFe_2_S_3_ (Fig. 2a) have been performed by a galvanostatic cycling at C/20/Li per formula unit (f.u.) in the potential window 0.5–3.0 V *versus* Li^+^/Li. Starting from our material, the first discharge is fragmented in a series of two main processes happening between 1.80 and 0.50 V. The first one is a slope (*A*) from 1.80 to 1.50 V, it is attributed to the insertion of one lithium into CuFe_2_S_3_ through a solid solution, accordingly to literature.^15^ This insertion follows the eqn (1):

1 CuFe_2_S_3_ + Li^+^ + e^−^ → LiCuFe_2_S_3_

**Fig. 2:**
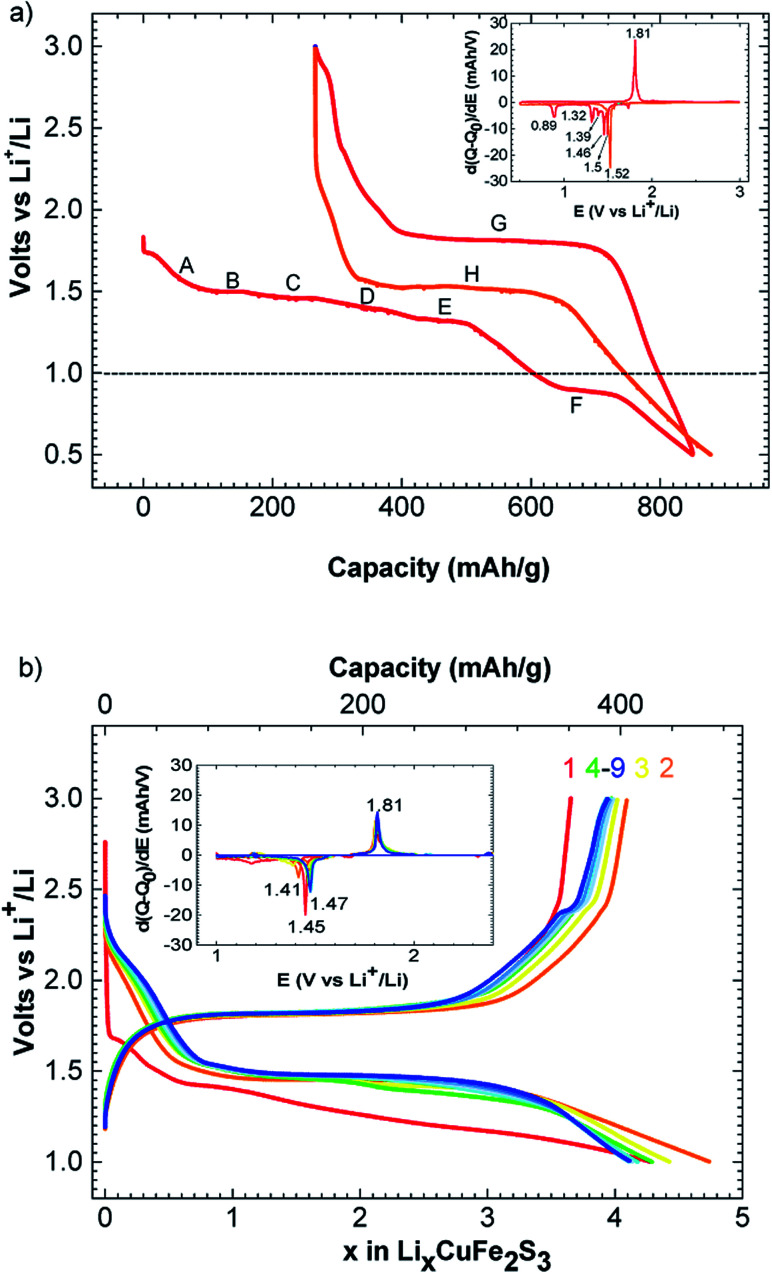
Voltage–composition curve for CuFe_2_S_3_ in the potential window 3.0–0.5 V at C/20/Li and inset: derivative curve |d*Q*/d*E*| *vs.* voltage (a); voltage–composition curve for CuFe_2_S_3_ in the potential window 3.0–1.0 V at C/2/Li and its corresponding derivative curve |d*Q*/d*E*| *vs.* voltage (b).

Note that the phase LiFeCuS_2_ has been reported^15^ and our sample is an intergrowth between chalcopyrite CuFeS_2_ and isocubanite CuFe_2_S_3_, a complete structural resolution of the lithiated phase is not possible. During the second process, we observe 5 plateaus at 1.50 (*B*), 1.46 V (*C*), 1.39 (*D*), 1.32 (*E*) and 0.82 (*F*) volt, respectively as shown on the derivative curve (inset Fig. 2a). These processes correspond to the reaction with 5 lithium and could then be assigned to copper and iron reduction to the metallic level as related in the case of Li/CuFeS_2_ system^15^ following the eqn (2):

2 LiCuFe_2_S_3_ + 5Li → Cu^0^ + 2Fe^0^ + 3Li_2_S

Because of the nature of our material (intergrowth of isocubanite and chalcopyrite), it is interesting to point out that only two domains are observed in the course of the first discharge of pure CuFeS_2_ phase (at 1.7 and 1.5 V, respectively^16^). Charging process occurs mainly through one plateau at 1.80 V (*G*) as shown on the derivative curve (Red curve, Fig. 2b). This plateau could be attributed to copper and iron oxidation leading to a mixture of cupper iron sulfides Cu_*x*_Fe_*y*_S_*z*_ (ref. 15, 23 and 24) and free sulfur formation. Consequently, this system becomes a hybrid between lithium ion and lithium sulfur battery. As observed on Fig. 2, the first discharge process is different than the second one where only one large plateau at 1.50 V (*H*) is observed. This can be due to SEI formation occurring at the same time than metal reduction in the first discharge. SEI formation has already been mentioned in similar conversion cathode study to be responsible for extra capacity like CuFeS_2_,^15^ Co_2_SiO_4_ (ref. 25) and CuCo_2_S_4_.^26^ In our case, an extra capacity of 260 mA h g^−1^ is observed. We particularly have to consider the presence of CuFeS_2_ in our material. CuFeS_2_ has been previously investigated and possesses similar electrochemical properties compare to CuFe_2_S_3_. Even if our material contains a large amount of CuFeS_2_, the electrochemical capacity cannot only be due to CuFeS_2_ activity. Therefore, this indicates that CuFe_2_S_3_ is an electroactive material.

A reversible capacity of 560 mA h g^−1^ (C/20/Li) is observed in the potential window of 1.52 V to 1.80 V. Please, note that an additional phenomenon appears below 1.50 V. This latter could correspond to different phenomena like electrolyte degradation or lithium insertion in the surface electrolyte interface as mentioned in previous report.^27,28^ We believe that the irreversible capacity is mainly ascribed to this slope and the SEI formation.

Study at a rate of C/2/Li and a cut off at 1.0 V are displayed Fig. 2b. The curve of C/2/Li shows a reversible and stable capacity of 400 mA h g^−1^ upon 9 cycles. Using this rate, a polarization of 340 mV is observed between formation and conversion of Cu_*x*_Fe_*y*_S_*z*_. This polarization is quite in accordance with previous electrochemical performance obtained for CuFeS_2_.^17^ We can notice that cutting off at 1.0 V improves the reversibility of the system. Note that the conversion process is not complete as last discharge plateau at 0.82 V is also cutted off. Consequently, first reduction plateaus appeared at lower voltage and then stabilized at 1.47 V after few cycles (Fig. 2b).

The intermittent galvanostatic titration (GITT) reported in Fig. 3a shows a biphasic process and allows us to access to the equilibrium potential in the course of the reduction with a thermodynamic potential of 1.65 V *vs.* Li^+^/Li. A polarization of 300 mV is observed. The potentiodynamic titration curve (PITT, Fig. 3b) reveals a bell-shape-type response on the reversible phenomenon, and confirms together with the sharpness of the peaks in the derivative curve (Fig. 2) that the reversible process is biphasic.

**Fig. 3:**
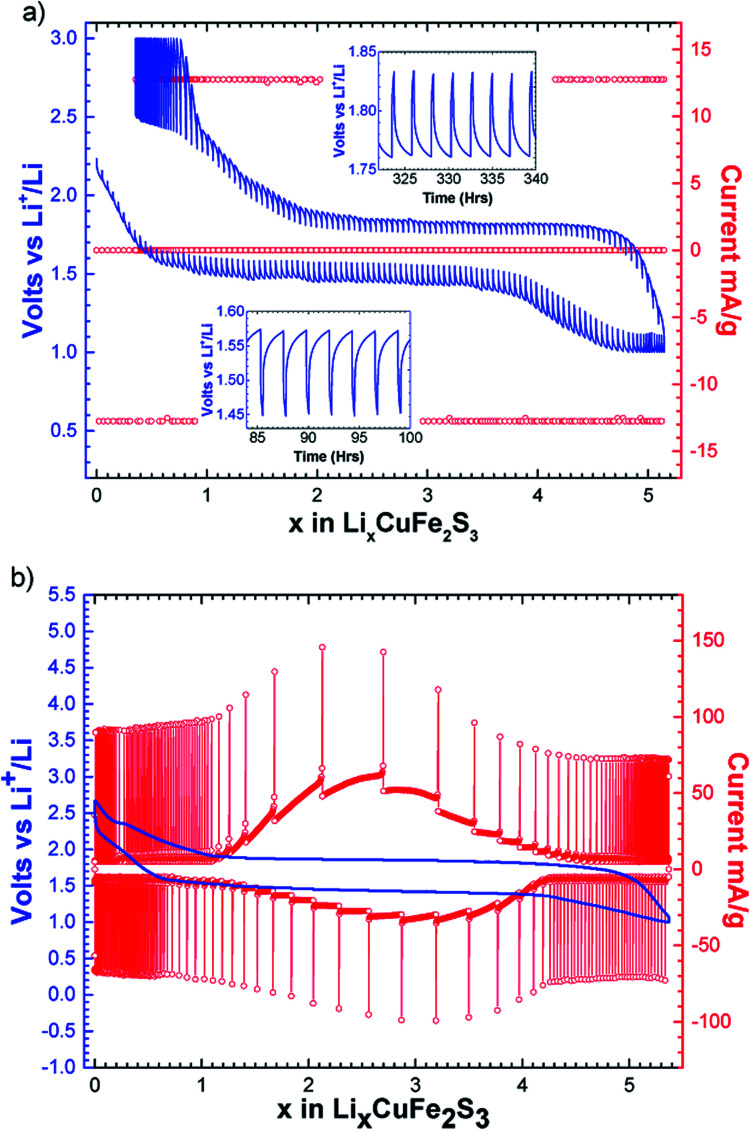
(a) Potential–composition curve of CuFe_2_S_3_ performed in a galvanostatic intermittent mode (GITT) with a rate of C/40 for 15 min and relaxation period of 2 h, (b) potentiometric titration curve (PITT) in the range of 3.0–1.0 V *vs.* Li^+^/Li using 5 mV potential step in duration of 1 h and current limitation equivalent to a galvanic current *I*_limit_ = *I*_C/100_.

To confirm the structural conversion occurring in the course of the electrochemical process, *ex situ* X-ray diffraction patterns have been recorded at the end of the first discharge and charge (Fig. 4). We can see on the discharge pattern that CuFe_2_S_3_ reflections disappeared (green middle curve, Fig. 4). Reflections on discharge pattern are attributed to copper (○), iron (Δ) and Li_2_S (+). After recharge (orange upper curve, Fig. 4), reflection close to CuFe_2_S_3_ and attributed to Cu_*x*_Fe_*y*_S_*z*_ (*) are observed among undefined products (□). This validates the conversion mechanism.

**Fig. 4:**
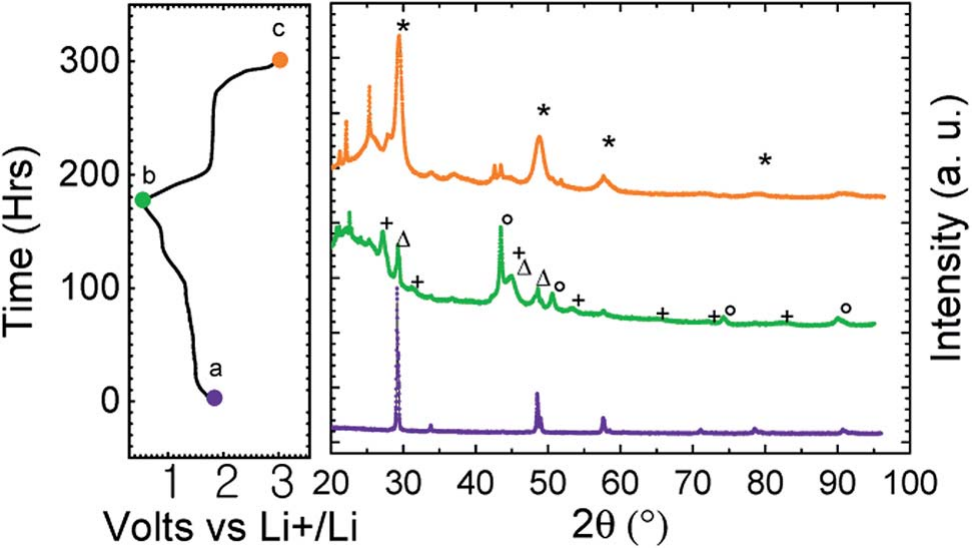
Powder X-ray diffraction patterns of (a) as prepared isocubanite CuFe_2_S_3_, (b) discharged phase down to 0.5 V (C/20) (c) charged phase up to 3.0 V (C/20). Cu_*x*_Fe_*y*_S_*z*_ (*), Li_2_S (+), Cu (○), Fe (Δ) and undefined products (□).

The specific capacity decreases with the increase in current density (Fig. 5a). The reversible capacity is about 425 mA h g^−1^ at the current density of C/5/Li and decreases down to 30 mA h g^−1^ at 10C/Li. When the current density is tuned back at C/5/Li, the specific capacity rebounds to 350 mA h g^−1^. This rate capability is comparable with previously reported CuFeS_2_ rate capabilities.^17^ Please note that we observed a capacity fading, which appeared to be not rate depending, and could be attributed to polysulfides formation known to be formed in lithium sulfur battery.^8^ Those polysulfides are the results of Li_2_S reduction that can form free sulfur.

**Fig. 5:**
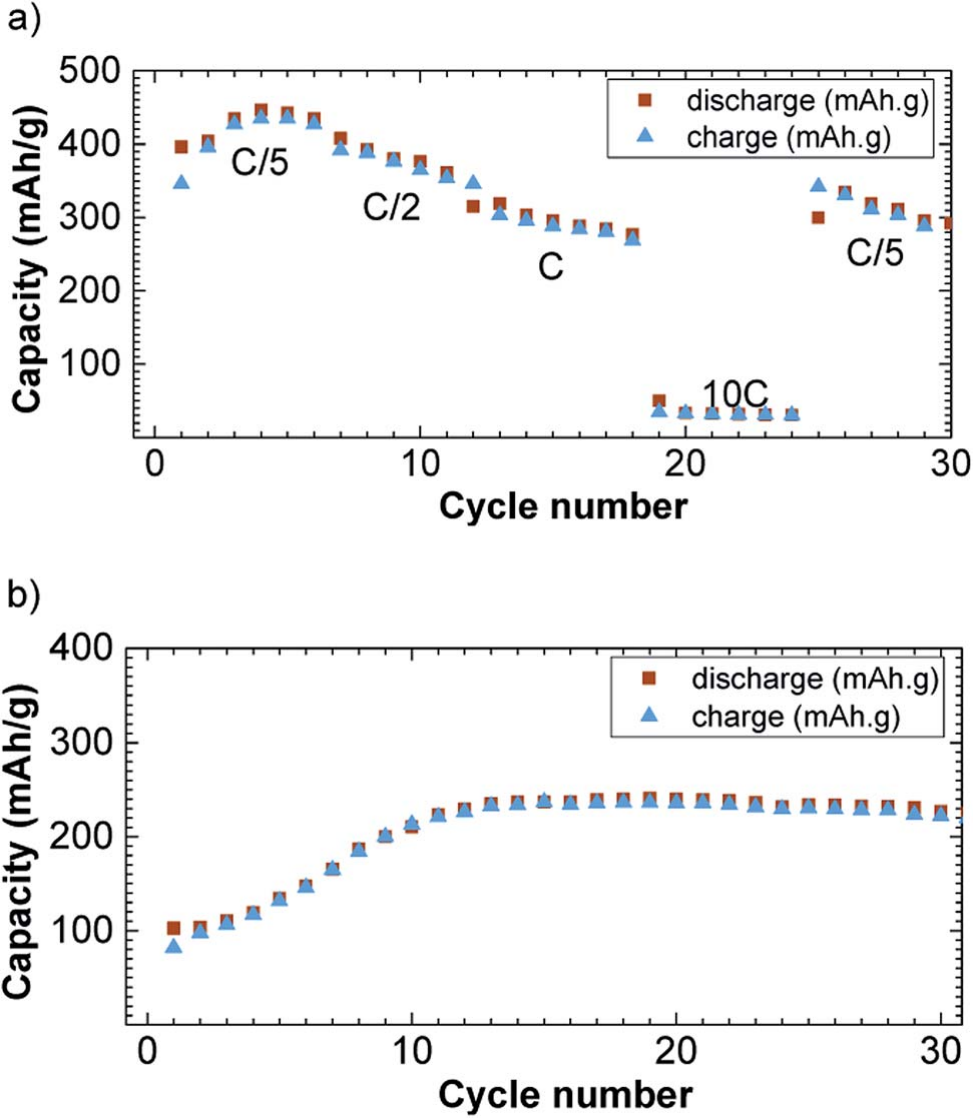
Rate (a) and cycling (b) capability *versus* cycle number at C/Li in 1.2–3.0 V potential window for the discharge (brown square) and charge (blue triangle) capacity *versus* cycle number.

On Fig. 5b, we have reported the cycling performances of CuFe_2_S_3_ at a current density of C/Li upon 30 cycles and with a cut off at 1.2 V. The cut off was necessary to avoid side reactions to occur as observed on the potential slope observed at the end of the first discharge. The capacity is rising in the first 10 cycles from 100 to 245 mA h g^−1^ and stabilizes around 250 mA h g^−1^. We believe this is due to the incomplete reaction together with side reaction despite the high voltage cut off.

The electrochemical behaviour of CuFe_2_S_3_ and CuFeS_2_ are relatively close to each other's. Their working potential is around 1.8 V and similar phenomena (conversion process, SEI formation, extra electrochemical capacity) are observed for both compounds. Furthermore, they are both semi-conductors. Concerning resistivity, our isocubanite sample (containing chalcopyrite) owns a resistivity of 0.7 mohm cm at 300 K while pure chalcopyrite possesses a higher resistivity at room temperature (measured between 20 and 200 mohm cm).^29^

## Conclusions

In this work, we demonstrate the conversion of the intergrowth between the two phases CuFe_2_S_3_ and CuFeS_2_ into Li_2_S and native copper and iron particles. Moreover, *ex situ* XRD at the end of the charge showed that a new phase Cu_*x*_Fe_*y*_S_*z*_ is formed. This new phase showed common diffraction peaks with the starting intergrowth but a complete structural resolution is not possible due to the low crystallinity of the material. More importantly, a reversible capacity of 425 mA h g^−1^ at a C/5/Li rate upon 10 cycles and with a cut off at 1.0 V is obtained. The redox potential of 1.65 V *vs.* Li^+^/Li gives an energy density of 600 W h kg^−1^. This result points out that despite the intergrowth nature of the material between isocubanite and chalcopyrite, we obtain comparable performance for this family of materials.

## Conflicts of interest

There are no conflicts to declare.

## Supplementary Material

## References

[cit1] Zhang K., Zhang T., Liang J., Zhu Y., Lin N., Qian Y. (2015). RSC Adv..

[cit2] Jache B., Mogwitz B., Klein F., Adelhelm P. (2014). J. Power Sources.

[cit3] Débart A., Dupont L., Patrice R., Tarascon J.-M. (2006). Solid State Sci..

[cit4] Han S.-C., Kim H.-S., Song M.-S., Lee P. S., Lee J.-Y., Ahn H.-J. (2003). J. Alloys Compd..

[cit5] Uetani Y., Yokoyama K., Okamoto O. (1980). J. Power Sources.

[cit6] Mukaibo H., Yoshizawa A., Momma T., Osaka T. (2003). J. Power Sources.

[cit7] Wang J., Wang G., Yang L., Ng S. H., Liu H. (2006). J. Solid State Electrochem..

[cit8] Cabana J., Monconduit L., Larcher D., Palacín M. R. (2010). Adv. Mater..

[cit9] Bonino F., Lazzari M., Rivolta B., Scrosati B. (1984). J. Electrochem. Soc..

[cit10] Chung J.-S., Sohn H.-J. (2002). J. Power Sources.

[cit11] Feng X., He X., Pu W., Jiang C., Wan C. (2007). Ionics.

[cit12] Choi J.-W., Cheruvally G., Ahn H.-J., Kim K.-W., Ahn J.-H. (2006). J. Power Sources.

[cit13] Siyu H., Xinyu L., QingYu L., Jun C. (2009). J. Alloys Compd..

[cit14] Kim S.-H., Choi Y.-J., Kim D.-H., Jung S.-H., Kim K.-W., Ahn H.-J., Ahn J.-H., Gu H.-B. (2008). Surf. Rev. Lett..

[cit15] Guo P., Song H., Liu Y., Wang C. (2017). ACS Appl. Mater. Interfaces.

[cit16] Ding W., Wang X., Peng H., Hu L. (2013). Mater. Chem. Phys..

[cit17] Wu X., Zhao Y., Yang C., He G. (2015). J. Mater. Sci..

[cit18] Wang Y., Li X., Zhang Y., He X., Zhao J. (2015). Electrochim. Acta.

[cit19] Fleet M. E. (1970). Z. Kristallogr..

[cit20] Pareek S., Rais A., Tripathi A., Chandra U. (2008). Hyperfine Interact..

[cit21] Barbier T., Berthebaud D., Frésard R., Lebedev O. I., Guilmeau E., Eyert V., Maignan A. (2017). Inorg. Chem. Front..

[cit22] Szymasski J. T. (1974). Acta Crystallogr..

[cit23] Son S.-B., Yersak T. A., Piper D. M., Kim S. C., Kang C. S., Cho J. S., Suh S.-S., Kim Y.-U., Oh K. H., Lee S.-H. (2014). Adv. Energy Mater..

[cit24] Li L., Cabán-Acevedo M., Girard S. N., Jin S. (2014). Nanoscale.

[cit25] Mueller F., Bresser D., Minderjahn N., Kalhoff J., Menne S., Krueger S., Winter M., Passerini S. (2014). Dalton Trans..

[cit26] Nie L., Wang H., Chai Y., Liu S., Yuan R. (2016). RSC Adv..

[cit27] Grugeon S., Laruelle S., Dupont L., Tarascon J.-M. (2003). Solid State Sci..

[cit28] Laruelle S., Grugeon S., Poizot P., Dollé M., Dupont L., Tarascon J.-M. (2002). J. Electrochem. Soc..

[cit29] Lefèvre R., Berthebaud D., Mychinko M. Y., Lebedev O. I., Mori T., Gascoin F., Maignan A. (2016). RSC Adv..

